# Personal

**Published:** 1917-02

**Authors:** 


### PERSONAL

Dr. Frank Brundage will move his residence and office to his new home, 572 West Ferry Street, March 1st.

Dr. Frank A. Vanente of Buffalo, a member of the hospital corps of the 74th Regt. has been commissioned as first lieutenant and assigned to the 3d Artillery (formerly 65th Infantry).

Dr. Louis J. Beyer has bought 449 Delaware Avenue, Buffalo.

Dr. George W. Cottis of Jamestown leaves early in Feb. for service in a base hospital of the British army in France, as a member of the Harvard unit.

Dr. Z. J. Lusk of Warsaw is in Florida, expecting to return about the middle of Feb.

Drs. S. N. Borowick, Leon J. Kurek and V. B. Beszczynski of Buffalo, have been appointed Camp Physicians of Kosciuszko Woodmen's Camp, No. 92.

